# Annotations

**Published:** 1904-03-05

**Authors:** 


### Annotations.

### The Optologist and the Pharmacist.

An optologist, it may be necessary to remind our readers, is the examined and certified product of a corporation boasting the proud title of the British Optical Association. He presents himself to the public as one having a thorough knowledge of general optics and a special knowledge of visual optics, and on the basis of this claim professes to be an expert in the treatment of errors of refraction. The spectacle-maker or optician, it appears, is on quite a different plane. He, honest man, must be content to grind his lenses in dull obscurity, and unblessed by that special knowledge of "visual optics" which is the distinguishing quality of those who have received the mystic designation conferred by the diploma of the B.O.A. Further, the optologist is, it seems, not only a new, but also a rare, phenomenon. From an address recently delivered by one of these superior beings, we learn that for the correction of the visual defects of the 42,000,000 of people found in the United Kingdom, there are only some 1,200 "competent optologists." It is indeed sad to learn on the same authority that the special opportunities for public service which the select 1,200 are competent to occupy are invaded by some 28,000 "spectacle sellers" who are ignorant of the elementary principles of refraction and impose on the public by the use of crude and obsolete methods of sight-testing. No wonder that in his zeal for the public interest the lecturer should call for the prohibition of all spectacle-selling except where the glasses are made "according to the prescription of an optologist." All this though new in form is hardly so in substance. The same kind of theory in essence can be heard in the booth of any travelling quack at a country fair. But it is surprising to find it welcomed by an association of pharmacists. Still more surprising is it to read that one member of the association regards sight-testing as a "special case of first aid" and another (a member also, if we mistake not, of the Pharmaceutical Council) recommends it as "a most appropriate side-line for a chemist." Members of the public of course have a right to entrust the care of their eyesight to whom they will. Some may select an automatic machine at a railway station, whilst for others an "optologist" may exercise a special charm. So far as we know there is no proposal to restrain by legal measures either of these delights of freedom. But one hardly expects to find that either one or the other should receive a recommendation from a respectable body of expert pharmacists. These, from their special experience, ought to have at least some appreciation of the responsibility incurred in the treatment of pathological conditions. They do themselves and their craft no credit when they suggest that such treatment may be undertaken by those who have no knowledge of these conditions.

### The Deaths in an Asylum.

The recent unfortunate occurrence of four deaths from chloral poisoning among the female inmates of the Portsmouth Asylum is an illustration of the dangers that must be run if the dispensing of powerful drugs is left in the hands of individuals who are not specially qualified for the purpose. From the evidence given at the inquest it appears that Dr. Marion Watson prescribed sleeping draughts containing chloral hydrate and potassium bromide for the four patients, and that the patients' medicine bottles were filled in the usual way from a dispensing bottle containing the mixture. The dispensing bottle was replenished from time to time from a stock bottle. But it is not clear that any particular individual was responsible for carrying out these simple dispensing operations. It was ascertained that the dispensing bottle had been empty shortly before the patients' bottles had been filled on the occasion in question, but it was not possible to discover who had refilled it. The borough analyst, Mr. E. Russell, deposed that he had analysed the contents of the dispensing bottle from which the draughts were taken, and of the unused draught bottle made up at the same time as those given to the deceased. The latter contained 384 grains of chloral hydrate, and could not have been taken from the dispensing bottle in its present state, while this in turn could not have been filled from the stock bottle, the contents of which he had also analysed. The worst feature of the case is that the immediate responsibility for the accident could not be fixed. If for some cogent reason a fully-qualified dispenser cannot be employed for the purpose of managing the asylum dispensary, the work should at least be in the hands of someone who can answer for any mishap that may occur. We strongly endorse the opinion expressed by the jury in this case that in future all dispensing should be performed only by fully-qualified persons.

### Taking the Oath.

The ceremony of taking the oath in the manner usually practised in the English Courts is one to which many persons entertain a decided objection. Yet few avail themselves of a method of escape open to all who care to utilise it. Even yet, therefore, it must be presumed it is not generally known that the practice of "kissing the book" is not compulsory. So long ago as 1888 an Act of Parliament was passed, giving to every witness who desires to exercise it the right to be sworn in the fashion prevalent in Scotland. All that the witness has to do when the oath is tendered to him is to claim this right, and, in accordance with the terms of the Act, "the oath shall be administered to him in such form and manner without further question." Neither the judge nor any other person has the least title to object or to cross-examine the witness on the matter. The procedure itself is simple in the extreme. It is certainly not wanting in solemnity and it entirely avoids the insanitary practice of kissing a book the possible previous experiences of which can hardly be contemplated with satisfaction. Members of the general public are apt to approach the witness-box with not unnatural trepidation and are disposed, therefore, to avoid anything which will emphasise the prominence of the position. Further, the great majority of them are unaware that any method of taking the oath other than the traditional one exists. In one of the divisions of the High Court it is true a notice has been posted announcing that any witness has the right to be sworn according to the Scottish fashion. But example is proverbially more potent than precept. Medical and other scientific witnesses might surely be expected to take the lead in the destruction of an uncleanly and possibly dangerous practice. By so doing they would not only protect themselves but would help to educate a public ever slow to learn better habits.

